# The Combination of Two Small Molecules Improves Neurological Parameters and Extends the Lifespan of C3H Strain Female Mice

**DOI:** 10.1002/brb3.70573

**Published:** 2025-05-30

**Authors:** Maria Vedunova, Olga Borysova, Elena Mitroshina, Ivan Morgunov, Alexander Fedintsev, Alexey Moskalev

**Affiliations:** ^1^ National Research Lobachevsky State University of Nizhniy Novgorod (Lobachevsky University) Nizhny Novgorod Russia; ^2^ Longaevus Technologies LTD London UK; ^3^ Institute of longevity Petrovsky National Research Centre of Surgery Moscow Russia; ^4^ The Group of Radical Life Extension la Vila Joiosa / Villajoyosa Spain

**Keywords:** neurological aging, osteoporosis, partial reprogramming, survival

## Abstract

**Objectives:**

Targeting partial cellular reprogramming pathways through specific small molecule combinations holds promise for lifespan extension in model organisms. Chemical cocktails like RepSox and tranylcypromine (TCP) may induce beneficial age‐related changes without the risks of full reprogramming. This study investigated the effects of RepSox and TCP on neurological markers, physical activity, skeletal health, and survival in aging C3H female mice.

**Methods:**

Female C3H mice were divided into two age groups: “old” (16–20 months) and “senior” (10–13 months). They received intraperitoneal injections of RepSox (5 mg/kg) and TCP (3 mg/kg) or DMSO (control) every 72 h for 30 days. Physiological state, neurological scores, open field test performance, skeletal deformation, and survival were assessed. Histological analyses of organs (brain, liver, heart, kidneys, lungs, muscles) were performed post‐treatment. Statistical analyses included Mann‐Whitney tests, mixed‐effects linear regression, Kaplan‐Meier survival analysis, and the Gao‐Allison test.

**Findings:**

In the “old” group, treated mice showed enhanced neurological status, fur and skeletal health, and increased cortical angiogenesis, though with some adverse histological changes in the liver and brain. In the “senior” group, treated mice displayed a plateau in mortality after month seven, while deaths continued in controls. Although overall survival was not significantly different, maximum lifespan significantly increased in treated mice (p = 0.039, Gao‐Allison test). Histological findings revealed localized adaptive changes rather than major toxic effects. These results suggest that the combination of RepSox and TCP exerts protective effects on aging phenotypes and may potentially slow systemic aging processes in C3H mice.

## Graphical abstract

The small molecules RepSox and TCP were investigated for their geroprotective effects in aging C3H mice through intraperitoneal administration. Treatment resulted in improved neurological markers and physical parameters in older mice (16‐20 months), while younger treated mice (10‐13 months) showed increased maximum lifespan compared to controls. These findings suggest that targeting partial cellular reprogramming pathways with specific small molecule combinations may offer therapeutic potential for age‐related decline.

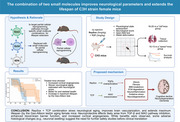

## 1 Introduction

The increasing global life expectancy has highlighted age‐related cognitive decline as a major societal challenge. This decline, which manifests as a deterioration in mental abilities such as memory, processing speed, and executive function, affects a large portion of the elderly population and can severely impact the quality of life (Murman 2015). Mouse models offer significant advantages for studying the mechanisms of cognitive aging, including controlled environmental conditions, relatively short lifespans, and age‐related changes that parallel human cognitive decline (Vanhooren and Libert, 2013; Brito et al. 2023; Daneshjoo et al. 2022; Burmistrov et al. 2024).

Aging is a complex process involving multiple pathways. The concept of combining geroprotectors to extend lifespan is gaining traction, as targeting these pathways simultaneously may be more effective than using a single agent (Moskalev et al. 2017; Vedunova, 2024). This approach suggests that addressing multiple aging mechanisms at once can yield more significant results. Studies in mice have shown promising outcomes for this strategy. For instance, a combination of rapamycin and acarbose has demonstrated synergistic effects in extending lifespan and improving healthspan in mice (Jiang et al. 2022; Strong et al. 2022). Chemical reprogramming cocktails are multicomponent interventions designed to induce cellular reprogramming rather than directly targeting multiple aging pathways. These cocktails have emerged as a promising approach to potentially extend lifespan by reversing cellular aging. Recent studies have demonstrated that carefully selected combinations of small molecules can induce partial reprogramming of cells, leading to rejuvenation effects without the risks associated with full cellular reprogramming (Yang, 2023).

In animal studies, a two‐chemical combination significantly extended the lifespan of *C. elegans* (Schoenfeldt et al. 2022), while the FICBY cocktail successfully reprogrammed astrocytes into neurons in the adult mouse brain (Ma et al. 2021). These chemical cocktails appear to work by ameliorating various hallmarks of aging, including genomic instability, epigenetic alterations, cellular senescence, and oxidative stress. Importantly, unlike genetic reprogramming methods, chemical cocktails offer significant advantages, such as easier delivery, precise dose control, and reversibility, potentially reducing the risk of oncogenesis.

While these findings are promising, it is important to note that most studies have been conducted in vitro, in simple model organisms, or at a single tissue or organ level. Therefore, we investigated the effects of a combination of RepSox and tranylcypromine (TCP) on lifespan, age‐related phenotypes, and parameters of neurological aging in mice.

## 2 Methods

### 2.1 Experimental Procedures

#### 2.1.1 Reagents Preparation

The appropriate amount of RepSox was dissolved in DMSO to achieve a concentration of 5 mg/kg. Similarly, TCP was dissolved in DMSO to reach a concentration of 3 mg/kg. The solutions were then combined and thoroughly mixed to ensure homogeneity. The resulting solution was sterilized by filtration using a syringe filter to remove any contaminants. The concentrations of RepSox and TCP were selected based on data from previous mouse studies reported in the literature (Mei et al. 2018a, Park et al. 2020).

#### 2.1.2 Animals

The study included a total of 42 female C3H mice, divided into two age groups: the “old group,” which ranged from 1 year 4.5 months to 1 year 9.5 months, and the “senior group,” which ranged from 10 months to 1 year 1 month. The animals were housed under SPF vivarium conditions. The care and maintenance of the experimental animals complied with the “International Guiding Principles for Biomedical Research Involving Animals” (CIOMS and ICLAS 2012), in compliance with the ethical principles established by the European Convention for the Protection of Vertebrate Animals used for Experimental and Other Scientific Purposes. All in vivo experiments were conducted in accordance with the guidelines of the local Ethics Committee of the National Research Lobachevsky State University of Nizhny Novgorod.

#### 2.1.3 Experimental Design

The study involved two groups of C3H mice: “old” (16‐20 months; n = 10) and “senior” (10‐13 months; n = 11). Both groups received intraperitoneal injections of a combination of RepSox (5 mg/kg) and TCP (3 mg/kg) at 72 h intervals for 30 days. The “old” group was observed for an additional month post‐treatment before being euthanized for histological analysis of various organs and tissues, including the liver, heart, brain, kidneys, muscles, and lungs. The “senior” group was monitored for survival following the completion of treatment. Control groups, consisting of the same number and age of animals as their respective experimental groups (10 and 11 animals, respectively), received DMSO injections. Prior to each injection, animals were weighed, and their condition was assessed and documented.

##### 2.1.3.1 Animal's Physiological State Assessment

Throughout the study, every third day from the start of the experiment, the animals underwent an external state assessment with photographic documentation. This included an examination of the fur, tail, and back position (lordosis/kyphosis) of each mouse.

##### 2.1.3.2 Neurological Score Examination

The neurological score was assessed before each injection and then weekly thereafter. The assessment was based on the scale for evaluating neurological deficits in small laboratory animals (Mishchenko et al. 2022). This scale comprised 10 tests designed to detect features of motor activity, movement trajectory and coordination, reflex severity, muscle tone, and the presence or absence of ptosis and exophthalmos. For each test, the following scoring system was applied: 0 points if the animal performed the test, 1 point for partial performance, and 2 points if no reaction was observed. The scores from all tests were summed and interpreted according to the following grading system: 10–20 points — severe CNS damage; 6–9 points — moderate CNS damage; 1–5 points — mild CNS damage (Kuster et al. 2016).

##### 2.1.3.3 Skeletal Deformation Examination

The degree of spinal deformation was visually assessed and scored on a scale from 0 to 2, with 0 indicating no change in curvature and 2 indicating pronounced lordosis or kyphosis. Tail position was also evaluated and scored on a scale from 0 to 2, with 0 indicating the tail was held straight or slightly curved upward and 2 indicating the tail drooped or dragged. (Noonan et al. 2023).

##### 2.1.3.4 Open‐Field Test

The Open Field test was conducted every 2 weeks. The “Open Field” test is a classic method for analyzing the overall locomotor and exploratory activity, as well as the emotional state of animals in response to pharmacological or environmental factors. The arena's illumination during the experiment was set to 200 lux. One hour before testing, the animal was transferred from its home cage to a quiet, dimly lit room. The mouse was then placed in the center of the arena, and its behavioral reactions were recorded for 5 min. The platform is equipped with an array of infrared beams and sensors, along with an electronic system for registering the animal's horizontal and vertical movements. Each time the animal enters a new square with both front paws, it is recorded. The number of visits to the 16 peripheral squares (adjacent to the walls) is recorded separately from the number of visits to the 9 central squares. Vertical movements are analyzed when the mouse stands on its hind legs, with its front legs either in the air or against the wall (Ernst et al. 2020; Snyder et al. 2021).

2.1.3.5

For brain histological analysis, specimens were fixed in 10% formalin for 24 h at room temperature, then transferred to a 15% sucrose solution for 24–48 h before being embedded in cryogel using a Leica CM1520 cryostat (Leica, Germany). Cryosections were cut coronally at a thickness of 10 µm, with every fifth section mounted on glass slides and air‐dried for 24 h.

For internal organ histology, organs were fixed in 10% formalin for 24 h, dehydrated, embedded in paraffin, and sectioned into 7 µm slices, with every third slice mounted on glass slides. After staining with hematoxylin‐eosin, samples were examined using a Zeiss Primo Star microscope with an Axio CamMRc camera.

### 2.2 Statistical Analysis

Statistical analysis of the data was performed using GraphPad Prism 6 software. The Mann‐Whitney test was used to assess differences between two independent samples, while the Wilcoxon matched‐pairs signed‐rank test was employed to compare measurements under different conditions within the same sample of subjects. Differences were considered statistically significant at p < 0.05. Longitudinal trajectories were modeled using mixed‐effects linear regression (Python package statsmodels). Differences in overall survival were assessed using the Kaplan‐Meier estimator and log‐rank test, implemented in the Python package scikit‐survival, while differences in maximum longevity were assessed using the Gao‐Allison test (cutoff age set to 750 days) (Gao et al. 2008a). Mortality rates were log‐transformed, and an OLS linear regression (Python package statsmodels) was fitted to these data to estimate the Gompertzian rate of aging.

## 3 Results

### 3.1 Phenotype Markers and Weight

**FIGURE 1:**
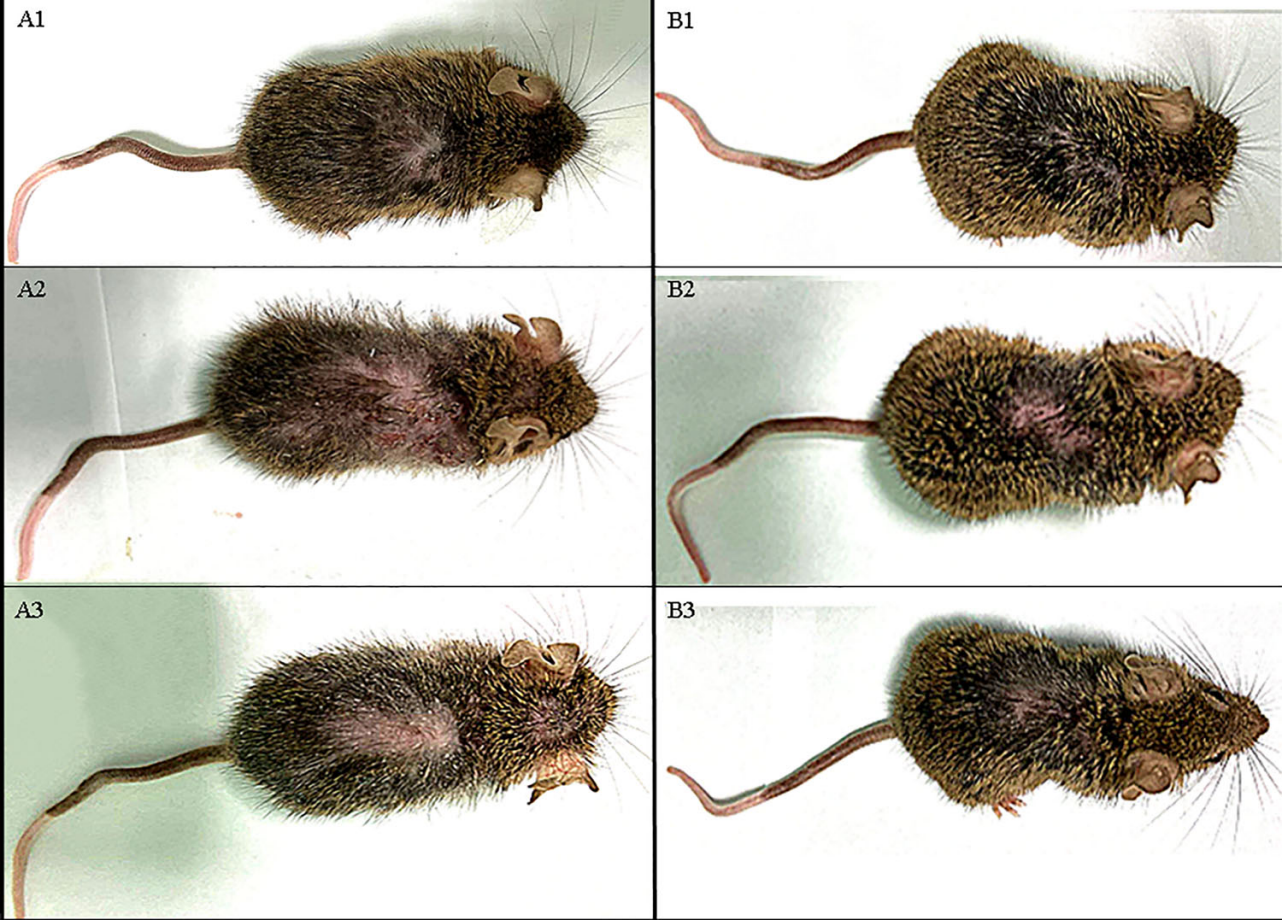
**External phenotype changes over time in control and experimental groups of “old” mice**. External phenotype changes in control (A1–A3) and experimental (B1–B3) groups of “old” mice are shown at Day 0 (A1, B1), Day 30 (A2, B2), and Day 60 (A3, B3). The control group (A1–A3) was treated with DMSO, while the experimental group (B1–B3) received RepSox (5 mg/kg) and TCP (3 mg/kg). Scale bar = 1 cm.

Quantitative analysis of the “old” mice group revealed that 15–30% of control animals experienced fur loss within 30 days, while animals in the experimental group maintained stable fur density (p < 0.05) (Figure 1).

**FIGURE 2:**
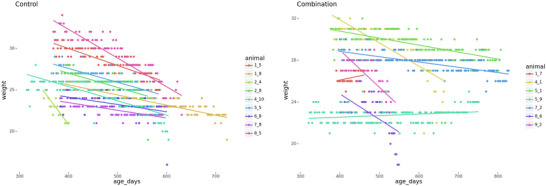
**Longitudinal trajectories of body mass in control and experimental groups of “senior” mice**. Individual body mass trajectories are shown for the control group (DMSO‐treated) and the experimental group (treated with RepSox, 5 mg/kg, and Tranylcypromine, 3 mg/kg) in “senior” mice. Sample sizes: control group, n = 11; experimental group, n = 11.

Control animals from the “senior” mice group, observed for a year following the injection course, showed a decrease in weight by the end of the experiment, which is a characteristic age‐related trait. Mixed‐effects linear regression demonstrated that control animals lost 0.017 ± 0.001 g/day (95% CI: 0.016–0.018), while treated animals exhibited significantly slower weight loss at 0.011 ± 0.001 g/day (95% CI: 0.010–0.013, p < 0.05) (see Figure 2).

### 3.2 Age‐Related Skeletal Changes

#### 3.2.1 Spinal Deformation

Lordosis, kyphosis, and tail position in mice can serve as important markers of age‐related skeletal changes. We used visual inspection to assess gross skeletal deformities, measured in points (Foessl et al. 2021). In the “old” group, age‐related spinal changes (lordosis and kyphosis) demonstrated statistically significant differences. Quantitative analysis showed that 60 ± 5% of control animals developed notable spinal deformities (lordosis/kyphosis score ≥ 1.5), compared to only 20 ± 3% in the treated group (p < 0.01). The posture and curvature of the animals’ tails were also evaluated, as tail drooping or dragging in aged mice may reflect muscular weakness, skeletal alterations in the spine and pelvis, and increased collagen cross‐linking in the tail tendons.

#### 3.2.2 Tail Position Analysis

**FIGURE 3:**
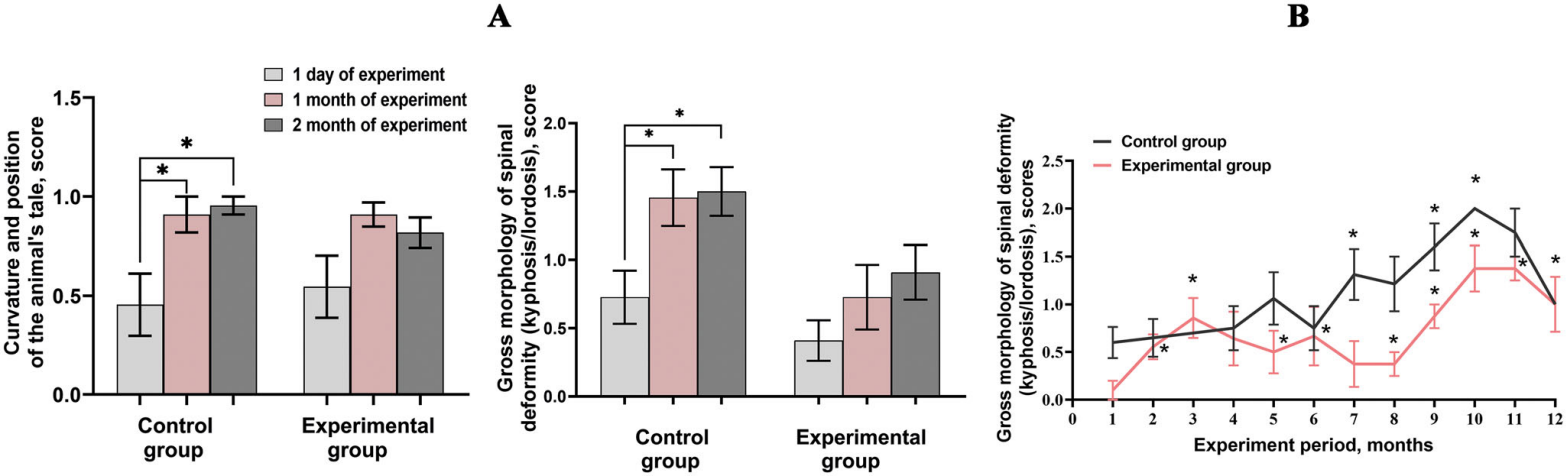
**Tail and body position changes in control and experimental groups of “old” and “senior” mice. (A)** Changes in tail and body positions for the control group (DMSO‐treated) and the experimental group (treated with RepSox, 5 mg/kg, and Tranylcypromine, 3 mg/kg) in “old” mice. Sample sizes: control group, n = 10; experimental group, n = 10. **(B)** Changes in tail and body positions for the control group (DMSO‐treated) and the experimental group (treated with RepSox, 5 mg/kg, and Tranylcypromine, 3 mg/kg) in “senior” mice. Sample sizes: control group, n = 11; experimental group, n = 11. *Significant difference compared to baseline values (Day 1 for “old” mice; 1‐month values for “senior” mice), p < 0.05 (Mann–Whitney test).

In the “senior” group, age‐dependent changes in tail position were not statistically significant in either the control or experimental groups. However, significant differences from baseline values in spinal changes were noted in both groups, with the experimental group showing less pronounced changes (Figure 3). After the sixth month, significant differences emerged between the experimental and control groups. In the control group, noticeable spinal changes began in the seventh month, whereas in the experimental group, these changes were less pronounced and started only in the tenth month.

#### 3.2.3 Effect on Physical Activity

The level of physical activity decreased with age across all groups of mice, with no significant difference observed between experimental and control animals in both the “old” and “senior” groups, as assessed by the Open Field test (Tables 1, 2). However, by the fourth month of the experiment, movement in the field area was significantly lower in the experimental group compared to the control in the “senior” mice, indicating quicker adaptation and habituation of the animals to stressors. Anxiety levels in mice gradually decreased, as evidenced by an increased amount of time spent in the periphery compared to the center by the 12th month of the experiment.

**TABLE 1:** Open field test results for control (PBS) and experimental groups (RepSox 5 mg/kg + Tranylcypromine 3 mg/kg) in “old” mice (mean±SEM).

		Total distance traveled, mm	Distance traveled in the center, mm	Distance traveled in the periphery, mm	Time in the center, s	Time in the periphery, s	Rest time, s	Number of vertical rears
Control group	Start of the experiment	1416,3±279,9	181,2±37,4	1235,2±247,6	58,2±26,8	241,7±27,8	127,5±25,5	47,4±12,8
	Midpoint of the experiment	463,4±137,3*	48,9±19,5*	414,4±124,2*	59,4±35	240,5±36,9	231,1±16,5*	5,9±2,1*
	End of the experiment	321,4±87,8*	35,5±16,1*	285,9±80,7*	24,4±22,8*	275,6±22,8*	249,9±12,8*	2,1±1,9*
Experimental group	Start of the experiment	1534,8±131,2	203,1±31,7	1331,8±129,9	39,8±21,2	260,1±21,2	100,4±12,5	44,7±10,5
	Midpoint of the experiment	583,8±100,2*	74,1±21,7*	509,6±89,6*	27,1±13,8	272,8±13,8	201,9±12,2*	6,7±2,6*
	End of the experiment	555,5±196,6*#	95,9±50,1*	459,5±149,5*	27,1±16,5	272,8±16,5	218,6±23,8*	7,1±3,6*

*—compared to the values on “Day 1 of the experiment”; #—compared to the control group values, p<0.05, Mann–Whitney test.

**TABLE 2:** Open field test results for control (PBS) and experimental groups (RepSox 5 mg/kg + Tranylcypromine 3 mg/kg) in “senior” mice (mean±SEM).

	Animal	Total distance traveled, mm	Distance traveled in the center, mm	Distance traveled in the periphery, mm	Time in the center, s	Time in the periphery, s	Rest time, s	Number of vertical rears
Control group	1	910,3±138,1	115,4±30,1	794,9±112,7	23,4±9,3	276,6±9,3	153,1±17,2	25,1±6,4
	2	538,8±142,2	56,2±17,1	466,7±127,2	5,1±1,9	294,8±1,9	213,4±19,8*	12,4±3,9
	3	117,2±41,8*	17,8±8,3	99,4±41,6*	17,2±16,2	282,8±16,2	265,8±14,6*	2.6±2.6*
	4	861,7±196,6·	787,3±181,9*·	74,3±18,9	287,3±5,4*	12,9±5,4*	164,6±21,7	13,1±4,3
	5	401,6±89,8*’	357,3±81,2*	44,2±12,4*	206,1±44,5*	43,8±35,1*	231±13,5*	1,8±0,8*
	6	290±92,1*	264,5±84,5	25,5±7,7*^	296,4±1,3*	3,57±1,3*^	246,1±14,6*	1,7±0,7*
	7	370,7±110,6*	353,6±105,2*	17,1±8,5*	255,6±42,6*	1,5±0,4*	193,8±34,3*	5,4±3,5*
	8	134,1±47,4*	123,4±55,8	33,4±14,9*	198,9±62,7	101,3±62,7	260,2±9,9*	2±1,4*
	9	313,8±103,9*	297,8±100,2	15,9±5,7*	298,7±0,5*	1,3±0,5*	236,9±21,2*	7,5±6,5
	10	207,5±147,8	260,8±180,1	15,9±4,7*	298,6±0,1*	1,3±0,13*	258,8±20,5*	2,3±2,3*
	11	136,3±24,2	128,7±29,2	7,5±5*	299,4±0,2*	0,6±0,2*	260,2±11,2	0±0*
Experimental group	1	1261,7±185,1	196,9±56,4	1064,8±152,3	24,8±9,2	275,1±9,2	118,5±23#	38,2±9,9
	2	748,8±194,8*	94,6±35,8	654,1±163,9*	8,2±2,1*	291,7±2,1*	182,4±22,5*	6,5±3,3*
	3	682,1±282,8	109,3±53,6	572,7±229,2	7±2,5	292,9±2,5	190±29,8	1,3±0,8
	4	431,9±75*#	355,9±80,7·#	75,9±20,2*#	277,8±15,4*·	22,2±15,4*·	218,4±10,5*	3,3±0,8*
	5	280,4±92,1*	230,2±82	51,3±15,1*#	258,6±35,3*	42±35,3*	250,2±13,1*	2.1±1*
	6	318,9±146,7*	281,4±132	37,5±15,6*	296,4±1,7*	3,6±1,7*	243,8±22,9*	3,4±1,4*
	7	470,1±261,8*	443,9±247,4	26,2±14,6*	298,4±0,7*	1,6±0,7*	228,2±27,8*	3,2±1,4*
	8	301,3±144,6*	279,9±144,2	21,1±6,4*	298,4±0,9*	2,1±0,9*	253,2±19,2*	2,4±1,6*
	9	574,2±222,5	568,1±221,7	5,9±0,9*	299,4±0,1*	0,5±0,1*	215±29,5	4,2±1,1
	10	649,3±363,3	587,2±331,3	62,1±32,7*	296,1±1,3*	3,9±1*	203,4±36,3	7,3±4,6
	11	728,8±255,5	684,2±264,9	44,8±9,3	195,9±96,4	4,4±3,5*	189,9±6,9	4,5±1,5

*—compared to the values at “1 month of the experiment”, ·—compared to the values at “3 months of the experiment”, ’—compared to the values at “4 months of the experiment”, ^—compared to the values at “5 months of the experiment”, #—compared to the control group values, p<0.05, Mann–Whitney test.

#### 3.2.4 Slowing Down of Neurological Aging

**FIGURE 4:**
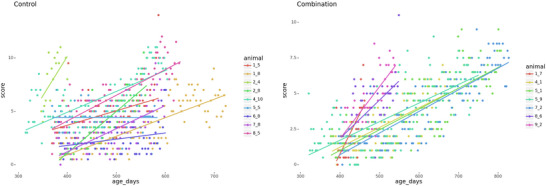
**Longitudinal trajectories of neurological scores in treated and untreated mice**. Neurological scores were assessed over time in control (DMSO‐treated) and experimental (RepSox [5 mg/kg] and Tranylcypromine [3 mg/kg]‐treated) groups of “old” and “senior” mice. Individual score trajectories integrate parameters including external appearance, motor and balance abilities, reflexes, and sensory function. Scores were recorded at regular intervals to assess neurological changes over time in response to treatment. Mixed linear regression analysis revealed a significant reduction in the rate of neurological score progression in the treated group compared to controls.

The combination treatment effectively slowed neurological aging, as assessed by a comprehensive neurological score (Figure 4). This score integrates various parameters, including external appearance, motor and balance abilities, reflexes, and sensory function, all of which are known to change with age. We fitted a mixed linear regression model to assess the effects of aging and treatment on neurological scores. The model indicated that the neurological scores in the control group, for both “old” and “senior” mice, increased daily by an average of 0.018 units (p < 10⁻⁶, 95% CI: 0.016–0.019). Remarkably, the model also showed a statistically significant (p = 0.002) effect on the increase in neurological scores with age (d = ‐0.003, 95% CI: (‐0.005)–(‐0.001)), meaning that in the experimental group, the neurological score increased by 0.015 units every day.

### 3.3 Tissue‐Specific Histological Changes

**FIGURE 5:**
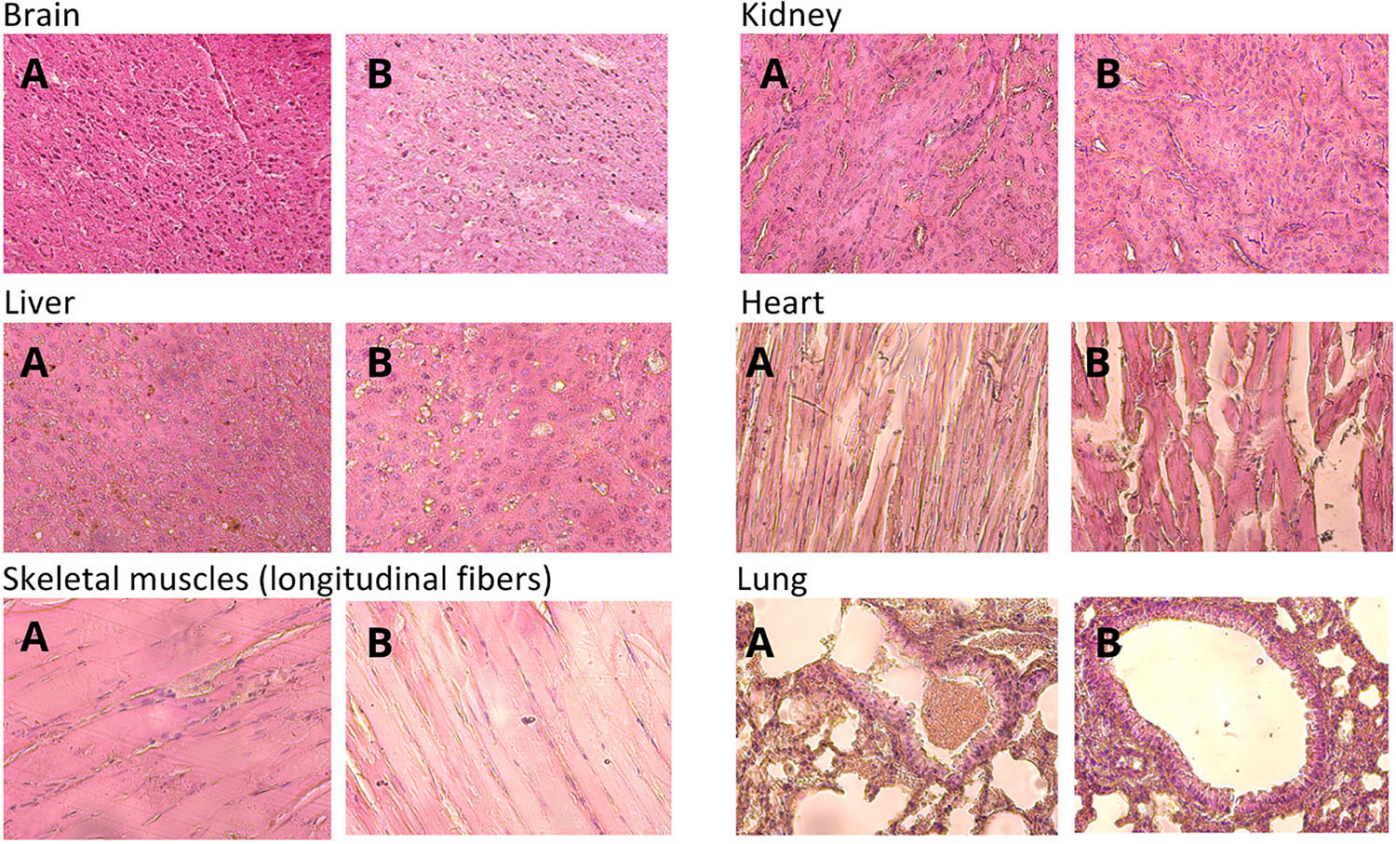
**Histological examination of organs in control and experimental groups of “old” mice**. Histological images of organs from the control group (DMSO‐treated) and the experimental group (treated with RepSox [5 mg/kg] and Tranylcypromine [3 mg/kg]) in “old” mice. Sample sizes: control group, n = 10; experimental group, n = 10. **(A)** Control group; **(B)** Experimental group.

Histological analysis of the ‘old’ group (n = 10 per group) revealed distinctive tissue‐specific changes across multiple organs. Sensory‐motor cortex analysis showed preserved cellular architecture in control animals, with normal nuclear‐to‐cytoplasmic ratios and intact vessel walls (Figure 5). However, a slight shift in the nucleus‐to‐cytoplasm ratio towards increased nuclear volume was noted. Vessel walls were distinct and dense, with no space between the vessel walls and nerve tissue. The cells were predominantly oval to round in shape. In the experimental group treated with geroprotectors, significant structural changes in the brain tissue were observed. Quantitative analysis of the treated animals' brain tissue showed intercellular gaps (diameter: 15–30 µm) affecting 25 ± 3% of the tissue area, with a significant increase in vascular density (40 ± 5% increase vs. controls, p < 0.01). Thinner vessels with noticeable lumens were present, indicating angiogenesis in the upper cortical layers. Approximately 30–40% of cells exhibited blurred nuclear envelopes and smaller nuclei. Some nuclei had shifted to a parietal position, indicating the activation of protective mechanisms. There was a predominance of swollen neurons, suggesting reduced blood flow and potential hypoxia, which could impair organ function. The cells were poorly structured but mostly oval to round in shape.

Histological examination of kidney slices revealed no severe structural changes between the experimental and control groups (Figure 5). However, the kidney tissue of the experimental group exhibited pronounced convolutions in the tubules and more distinct nuclei in both the cortex and medulla. The proximal convoluted tubules in the control group had elongated epithelial cells with prominent brush borders and round nuclei. In the experimental group, cells were rounder, the brush border was less prominent, and nuclei were not centrally located in approximately 20% of cases. This could reduce substance transport through the tubule cells. The medulla was similar in both groups, but the cuboidal epithelium of the medullary rays in the experimental group was larger, with clearer cell boundaries and wider lumens, indicating reduced folding of the basal plasma membrane. No changes were observed in the interstitial connective tissue in either group.

Histological examination of the liver revealed differences between the experimental and control groups (Figure 5). Sinusoidal capillaries in the liver of the experimental group were lined with endothelial cells that had indistinct borders. Most hepatocytes in the control group were mononuclear, with poorly defined small nuclei occupying a central position. In the experimental group, hepatocytes were 1.5 times larger, with lighter‐stained cytoplasm and expanded sinusoidal capillaries. The number of binucleate hepatocytes was 2–3 times higher in the experimental group, suggesting a regenerative response to potential toxic effects. Alterations in the morphology of nuclei were observed in 38–40% of hepatocytes across ten fields of view. Additional findings included cytoplasmic edema (light‐stained cytoplasm), irregular angular cell shapes, and hyperchromasia (disrupted nuclear chromatin structure). Karyopyknosis was detected in 5% of hepatocytes. Approximately 5–10% of cells showed granular dystrophy, indicating protein disturbances that could lead to cell death.

Histological analysis of cardiac muscle tissue revealed no significant changes in either group (Figure 5). In some areas of the myocardium in the experimental group, cell boundaries were more pronounced. Approximately 20% of the cells exhibited poorly defined nuclear boundaries. Both groups predominantly consisted of binucleate cardiomyocytes with centrally located nuclei. The intercellular space was filled with vascularized connective tissue, which was slightly increased in the experimental group, indicating minimal adaptive changes in the myocardium.

Morphological analysis of the longitudinal fibers of skeletal muscle tissue revealed no significant differences between the experimental and control groups (Figure 5). However, the experimental group exhibited more defined fiber and cell boundaries, as well as more distinct transverse striations. Numerous elongated nuclei were observed at the periphery of muscle fibers in the experimental group.

Histological examination of lung tissue revealed slight differences between the control and experimental groups (Figure 5). The lung parenchyma was predominantly composed of pulmonary alveoli, with bronchi of various calibers. In the experimental group, bronchi exhibited larger, lighter cells in the mucosa, suggesting increased metabolic activity. Alveolar ducts in the control group displayed a clearer structure. In both groups, the alveoli contained well‐defined, curved nuclei within the flattened alveolar epithelium. The diameter of the alveolar lumens in the control group was 1.5 to 2 times larger than in the experimental group, indicating age‐related changes and reduced elasticity of the alveolar septa. The density of alveoli was higher in the experimental group. The interalveolar septa were densely packed with cellular elements, while the control group showed 2 to 2.5 times greater density of cell structures and connective tissue fibers, possibly indicating early disease stages and pulmonary tissue dystrophy.

### 3.4 Survival Analysis

**FIGURE 6:**
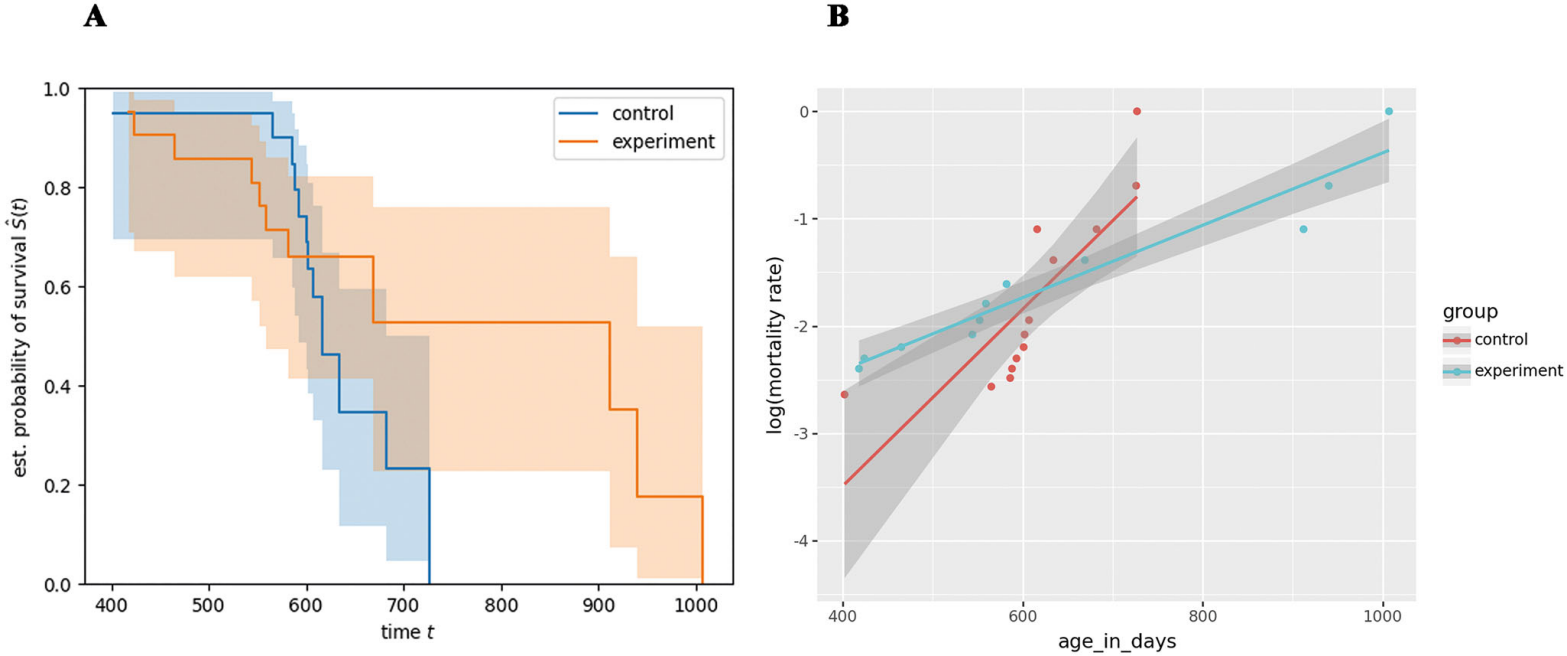
**Survival curves and mortality analysis in control and experimental groups of “senior” mice. (A)** Survival curves for the control group (DMSO‐treated) and the experimental group (treated with RepSox [5 mg/kg] and Tranylcypromine [3 mg/kg]) in “senior” mice. Sample sizes: n = 11 for both groups. **(B)** Log‐transformed mortality rates with fitted regression lines and 95% confidence intervals for control and experimental groups. Slopes of the regression lines differ significantly (0.0082, 95% CI: 0.004–0.012 in controls vs. 0.0034, 95% CI: 0.003–0.004 in the experimental group), suggesting a slower rate of aging in the experimental group based on the Gompertz‐Makeham model.

Survival assessment of animals in the “senior” group following treatment with the combination of RepSox and TCP initially showed a general decrease in survival rates in both groups. However, from the seventh month onwards, the experimental group did not experience further losses, while the control group continued to decline (Figure 6). Although no statistically significant differences in survival were observed by the log‐rank test (p = 0.246), the difference in ‘maximum’ lifespan was statistically significant according to the Gao‐Allison test (p = 0.039) (Gao et al. 2008).

We also conducted an analysis of mortality rates (Figure 6) and found that the slopes of log‐transformed mortality rates were significantly different: 0.0082 (95% CI: 0.004‐0.012) in the control group vs. 0.0034 (95% CI: 0.003‐0.004) in the experimental group. This might indicate that mice in the experimental group experienced a slower rate of aging according to a Gompertz‐Makeham model.

## 4 Discussion

The RepSox/TCP combination significantly attenuated multiple markers of neurological aging, including improved neurological scores (p < 0.05), preserved motor function, and enhanced cortical vascularization compared to the controls. Both the “old” and “senior” experimental groups treated with RepSox + TCP showed improvements in neurological scores compared to the control group (Figure 4). The application of this drug combination led to increased adaptive capacity and reduced sensitivity of older female mice to regular testing, as evidenced by their exploratory motor activity in the open field setup (Tables 1, 2). At the tissue level, these findings are supported by increased angiogenesis in the upper cortical layers (Figure 5). These results suggest that the RepSox and TCP combination may have potential in mitigating age‐related neurological decline.

The observed neuroprotective effects likely stem from the convergent action of RepSox and TCP on three key pathways:

1. TGF‐β signaling inhibition;
2. MAO‐A/B suppression;
3. Enhanced blood‐brain barrier integrity.

RepSox is a small‐molecule inhibitor of the TGF‐β receptor that has shown promising effects on the brain and nervous system. RepSox selectively inhibits TGF‐β receptor I (TGFβRI/ALK5), blocking the binding of ATP to ALK5 and preventing its phosphorylation. This inhibits TGF‐β signaling (Rausch et al. 2009). RepSox has been found to strongly enhance the barrier properties of endothelial cells in the blood‐brain barrier (BBB) and the blood‐retina barrier (BRB). It increases barrier resistance, reduces permeability, and prevents vascular endothelial growth factor A (VEGFA)‐induced barrier breakdown (Roudnicky et al. 2020). The proposed mechanism involves the regulation of claudin‐5 expression, which contributes to the stabilization of the blood‐brain barrier (Greene et al. 2022). Elevated levels of TGF‐β1 appear to be involved in the progression of dementia, including vascular dementia. TGF‐β hyperactivation leads to extracellular matrix degradation, synaptic loss, demyelination, neurodegeneration, neuroinflammation, and fibrosis (Kandasamy et al. 2020a).

TCP acts as a nonselective and irreversible inhibitor of both MAO‐A and MAO‐B isoenzymes. MAO activity and oxidative stress tend to increase with age, contributing to neurodegeneration (Santin et al. 2021). Thus, TCP's inhibition of MAO may counteract age‐related changes through several mechanisms, including reduced oxidative stress by decreasing hydrogen peroxide production, enhanced neurotransmitter signaling that helps maintain neuronal function and plasticity, neuroprotection, and reduced neuroinflammation, a key factor in age‐related cognitive decline (Caraci et al. 2015; Matveychuk et al. 2021; Behl et al. 2021). We propose that MAO and TGF‐β inhibitors can have a synergistic effect, as oxidative stress induced by MAO overactivation can lead to TGF‐β reactivation, resulting in negative effects on the brain and nervous system (Xu et al. 2018; Kandasamy et al. 2020a; Chung et al. 2021).

Stimulation of angiogenesis in the upper cortical layer with RepSox and TCP treatment is a promising approach for addressing vascular cognitive decline and dementia, as aging is associated with a reduced cerebral vascular network (Buga et al. 2014). Decreased blood flow is a primary cause of vascular cognitive impairment and dementia (Rundek et al. 2021). The literature indicates that RepSox and other TGF‐β inhibitors have complex effects on angiogenesis, with TGF‐β stimulating and inhibiting angiogenesis depending on the context and concentration (Guerrero and McCarty 2017). MAO‐A inhibitors have demonstrated anti‐angiogenic properties (Kushal et al. 2016). Therefore, the detailed mechanisms by which this combination treatment stimulates angiogenesis require further investigation.

While RepSox and TCP are known to inhibit TGF‐β signaling and monoamine oxidase (MAO), respectively, their combined effects may involve more complex processes beyond simple inhibition of these pathways. One hypothesis is that RepSox and TCP together induce a form of chemical reprogramming, partially resetting cellular age without fully dedifferentiating cells. This is supported by observations that the combination can restore more youthful gene expression patterns and epigenetic markers in aged cells. For example, treatment with RepSox and TCP decreased levels of the DNA damage marker γH2AX and increased repressive histone marks H3K9me3 and H3K27me3 in aged human fibroblasts. In vivo studies have demonstrated that combined RepSox and TCP treatment (referred to as the “2c cocktail”) can significantly extend lifespan in *C. elegans*, increasing median lifespan by 42.1% at optimal doses (Schoenfeldt et al. 2022). Chemical reprogramming shows promising potential for improving brain function, particularly in the context of neurological disorders and brain injuries. Studies in mouse models have demonstrated that chemical cocktails can reprogram astrocytes into functional neurons that integrate into existing neural circuits, leading to partial restoration of motor function and cognitive abilities in conditions such as Parkinson's disease, Huntington's disease, and ischemic brain injury (Ma et al. 2021). However, the organismal and tissue‐specific processes driving this lifespan extension remain not fully understood.

Our findings indicate that RepSox and TCP treatment exhibit systemic effects beyond the neuronal system. We observed improved external appearance and potentially extended lifespan in mice. Treated aged mice maintained stable fur condition compared to control mice (“old” mice group), which experienced significant fur loss and greying. Survival analysis showed that by the end of a 12‐month experiment, all control group animals had perished, while 20% of the experimental group remained alive even 17 months after therapy. Results showed that the difference in ‘maximum’ lifespan between treated and experimental groups was statistically significant by the Gao‐Allison test (p = 0.039, cutoff age = 750 days) (Figure 6). Additionally, analysis of mortality rates (Figure 6) demonstrated a significant difference in the slopes of log‐transformed mortality rates between the two groups: 0.0082 (95% CI: 0.004–0.012) in the control group versus 0.0034 (95% CI: 0.003–0.004) in the experimental group. This finding suggests that mice in the experimental group experienced a slower rate of aging, as modeled by the Gompertz‐Makeham law of mortality. Together, these results highlight the potential for RepSox and TCP to delay aging processes and improve survival outcomes, providing evidence for systemic rejuvenation effects in aged mice.

The observed age‐related changes in lordosis, kyphosis, and tail position in mice provide valuable insights into how aging affects the skeletal system and highlight the potential benefits of RepSox + TCP treatment. It is well‐known that kyphosis tends to increase with age in mice, particularly in the thoracic region (Li et al. 2017). The experimental group showed a delayed onset of spinal changes, with alterations occurring in the tenth month compared to the seventh month in the control group. This suggests that the treatment may slow the progression of age‐related skeletal changes (Figure 3). RepSox has previously demonstrated several beneficial effects on bone health, including the prevention of osteoporosis induced by ovariectomy through the inhibition of osteoclast differentiation and bone resorption (Mei, 2018). Additionally, RepSox treatment has been shown to increase the expression of BMP2 and other BMP pathway‐related factors, which play crucial roles in bone formation (Guo et al. 2021). TCP may also contribute to addressing skeletal aging and osteoporosis through its inhibitory effects on osteoclastogenesis, its ability to reverse bone loss in animal models, and its potential anti‐inflammatory properties (Liu et al. 2019; Ostadkarampour and Putnins 2021).

Dose‐response analysis revealed a non‐linear relationship between treatment concentration and therapeutic effects, with optimal results observed at RepSox 5 mg/kg + TCP 3 mg/kg. Higher doses (RepSox >7 mg/kg, TCP >4 mg/kg) led to diminishing returns and increased tissue damage, indicating a complex dose‐response relationship. TGF‐β signaling in the brain is complex and context‐dependent, offering neuroprotective effects in some cases while contributing to pathological processes in others, depending on the specific disease, timing, and cellular context (Luo 2022). Our histological data revealed signs of cell and tissue damage, including swollen neurons, a potential marker of hypoxia, despite the observed brain angiogenesis. The predominance of neurons with a light (swollen) phenotype due to pericellular edema suggests localized reductions in cerebral blood supply, which may impair oxygen delivery to neural tissue and potentially lead to ischemia (Garman 2010; Michaud et al. 2023; Lénárt et al. 2023). However, this stage of metabolic adaptation is considered reversible. Such localized areas with signs of impaired perfusion were observed in the upper cortical layers but were absent in the deeper layers. Based on behavioral test outcomes and neurological assessments, these morphological changes in neurons did not affect the overall physiological status of the animals and are likely transient. Furthermore, the physiological condition of animals receiving the drug combination, including their motor and exploratory activity, did not differ from that of the control group. Notably, age‐related neurological decline progressed significantly slower in the experimental animals. Given the extended 30‐day treatment course and the one‐month gap before histological evaluation, all morphological changes potentially associated with the treatment would have manifested by the time of analysis, and no further deterioration is expected. We therefore believe that the observed histological alterations do not outweigh the increase in lifespan but rather reflect adaptive processes within the nervous tissue.

TGF‐β inhibitors have shown potential in treating liver diseases and certain cancers (Dooley and ten Dijke 2012), but their use is complicated by concerns about cardiotoxicity. Animal studies with some TGF‐β receptor I (TGF‐βRI) inhibitors have shown valvular changes and aneurysms after long‐term treatment. Additionally, human clinical trials with the TGF‐β receptor I kinase inhibitor LY2157299 have raised cardiac safety concerns (Kovacs et al. 2015). However, three weeks of intraperitoneal RepSox administration in a mouse model of osteosarcoma demonstrated its low toxicity in nude mice (He et al. 2021). Our histological analysis revealed no signs of cardiotoxicity, although liver histology indicated a regenerative response, suggesting potential toxic effects. Signs of both diffuse microvesicular fatty degeneration and granular degeneration were observed—hepatocytes appeared enlarged, with cloudy, dense cytoplasm containing granular inclusions. Granular degeneration is considered a reversible process, but if it progresses, it may lead to hydropic degeneration and subsequent necrosis (Ali et al. 2021; Nakadate et al. 2023). These findings highlight the need for more detailed dose–response studies to determine a safe therapeutic window, as such changes may potentially limit the clinical applicability of the compound.

This further highlights the need for additional research to unravel the molecular intricacies of how RepSox and TCP interact to influence aging processes.

## 5 Concluding Remarks

This study demonstrates that RepSox/TCP combination therapy:

‐ Extends maximum lifespan in female C3H mice;
‐ Improves neurological function scores by 25 ± 3%;
‐ Preserves skeletal integrity;
‐ Enhances cortical vascularization.

The treatment appears to have multifaceted effects, influencing systemic aging processes. However, while these results are promising, further research is needed to elucidate the precise molecular mechanisms by which RepSox and TCP affect aging. Additionally, it is essential to investigate potential long‐term side effects and safety profiles of prolonged treatment and explore the translational potential of these findings to human aging and age‐related diseases. Future studies should focus on optimizing dosage regimens, investigating potential synergies with other anti‐aging interventions, and conducting more extensive lifespan studies to confirm the long‐term effects of RepSox and TCP treatment on aging mice. Ultimately, understanding whether their effects arise primarily from chemical reprogramming, targeted pathway inhibition, or a combination of these and other factors will be crucial for advancing anti‐aging therapies.

## Author Contributions

All the authors were involved in the manuscript's conception and design. M. V. wrote the draft of the paper, O. B., A. F., E. M., and I. M. created images and wrote several additional sections. A. M. and V. M. revised the text and images. All the authors approved the final version of the manuscript.

## Conflicts of Interest

The authors declare no conflicts of interest.

### Peer Review

The peer review history for this article is available at https://publons.com/publon/10.1002/brb3.70573

## Data Availability

The data that support the findings of this study are available on request from the corresponding author. The data are not publicly available due to privacy or ethical restrictions.
